# Type 2 diabetes and in-hospital complications after revision of total hip and knee arthroplasty

**DOI:** 10.1371/journal.pone.0183796

**Published:** 2017-08-24

**Authors:** Ana López-de-Andrés, Valentín Hernández-Barrera, Maria A. Martínez-Huedo, Manuel Villanueva-Martinez, Isabel Jiménez-Trujillo, Rodrigo Jiménez-García

**Affiliations:** 1 Medicine and Surgery, Psychology, Preventive Medicine and Public Health, Medical Microbiology and Immunology, Nursing and Oral Medicine Department. Universidad Rey Juan Carlos. Alcorcón. Comunidad de Madrid. Spain; 2 Preventive Medicine Department, Hospital Universitario la Paz. Madrid, Spain; 3 Unit of Revision Hip and Knee Arthroplasty. Unidad de Recambios Protésicos. Hospital Beata María. Madrid. Spain; Georgia Regents University, UNITED STATES

## Abstract

**Purpose:**

To assess the effect of type 2 diabetes (T2DM) on hospital outcomes such as in hospital postoperative complications (IHPC), length of hospital stay (LOHS) and in-hospital mortality (IHM) after the revision of total hip arthroplasty (RHA) and total knee arthroplasty (RKA) and to identify factors associated with IHPC among T2DM patients undergoing these procedures.

**Methods:**

We performed a retrospective study using the Spanish National Hospital Discharge Database, 2005–2014. We included patients who were ≥40 years old that had undergone RHA and RKA. For each T2DM patient, we selected a year-, gender-, age- and Charlson Comorbidity Index-matched non-diabetic patient.

**Results:**

We identified 44,055 and 39,938 patients who underwent RHA (12.72% with T2DM) and RKA (15.01% with T2DM). We matched 4,700 and 5,394 couples with RHA and RKA, respectively. Any IHPC was more frequent among patients with T2DM than among non-T2DM patients (19% vs. 15.64% in the RHA cohort and 12.94% vs. 11.09% in the RKA cohort, respectively). For patients who underwent RHA, postoperative infection (4.51% vs. 2.94%, p<0.001), acute post-hemorrhagic anemia (9.53% vs. 7.70%, p<0.001), mean LOHS and IHM were significantly higher in patients with T2DM. Among RKA patients, the incidence of acute posthemorrhagic anemia (7.21% vs. 5.62%; p = 0.001) and urinary tract infection (1.13% vs. 0.72%; p = 0.029) was significantly higher in patients with diabetes. Older age, obesity, infection due to internal joint prosthesis, myocardial infarction, congestive heart failure, mild liver disease and renal disease and emergency room admission were significantly associated with a higher risk of IHPC in T2DM patients. IHPC decreased over time only in T2DM patients who underwent RHA (OR 0.94, 95%CI 0.89–0.98).

**Conclusions:**

Patients with T2DM who underwent RHA and RKA procedures had more IHPC after controlling for the effects of possible confounders. LOHS and IHM were also higher among RHA patients with diabetes. Older age, comorbidity, obesity and emergency room admission were strong predictors of IHPC in diabetic patients.

## Introduction

Despite the effectiveness of total hip arthroplasty and total knee arthroplasty, over the first 5 years, approximately 6% of these procedures will need to be revised [1]. The demand for the revision of total hip arthroplasty (RHA) and total knee arthroplasty (RKA) are projected to grow by 137% and 601%, respectively, by 2030 [2].

RHA and RKA have higher complications rates than primary total joint arthroplasty [3–5]. Recently, a study that used the American College of Surgeon’s National Surgical Quality Improvement Program database presented complication rates of 7.4% for RHA and 4.7% for RKA [5]. Lakomkin et al. [6] concluded that greater comorbidity is a risk factor for postoperative mortality, major adverse events, minor complications, blood transfusions, and increased length of hospital stay (LOHS) following RHA.

Diabetes is a predictor of poor orthopedic surgical outcomes after joint arthroplasty that requires subsequent revision surgery [7–10]. King el al. [11] found greater rates for revision arthroplasty in the youngest (45 to 55 years for knee and hip) and oldest (65 years and over for knee) age groups compared to the non-diabetic cohorts. In a previous study, we found that the presence of diabetes was associated with poorer immediate postoperative outcomes after primary joint arthroplasty (higher risk of mortality and higher LOHS) compared to non-diabetic adults [12].

The study of the effect of diabetes on outcomes of revision joint arthroplasty is relevant for several reasons: i) The number of patients with diabetes and arthritis is increasing; therefore, the number of patients with diabetes electing to undergo primary and revision joint arthroplasty is likely to increase as well [2, 13]. ii) Comparisons of revision rates and outcomes in specific risk groups between countries may provide information that would assist in understanding the differences and aid in the planning of the provision of healthcare services [14]. iii) Data on the magnitude of this problem will help in the planning of future resources and optimizing indications and proper patient selection [6–8]. iv) Quantifying the possible negative effect of diabetes on outcomes would allow surgeons to advise patients specifically about these increased risks when obtaining informed consent and would encourage patients to be meticulous about their perioperative care [15]. v) Identifying modifiable risk factors that substantially contribute to poor clinical outcomes is critically important for the construction of predictive models in order to control these risk factors before proceeding with total joint arthroplasty.vi) More information will allow for better perioperative preparation and the correctly managed perioperative and postoperative care of patients with diabetes and will improve the cooperation of the orthopedist, dialectologist and anesthesiologist and the implementation of specialized departments to take care of these high risk patients. Specific perioperative interventions could include, among others, tight blood glucose control, the use of more efficient means to rehabilitate and discharge patients and an increased rate of discharges to short and long-term care facilities [16, 17]. However, to our knowledge, in hospital postoperative complications (IHPC) after RHA and RKA in patients with type 2 diabetes (T2DM) have not been studied in Spain.

The aims of our study are to use the Spanish National Discharge Database to i) assess the effect of T2DM on hospital outcomes such as IHPC, LOHS and in-hospital mortality (IHM) after RHA and RKA, and ii) to identify factors associated with IHPC among T2DM patients undergoing these procedures.

## Material and methods

We analyzed data from the Spanish National Hospital Discharge Database between January 1, 2005 and December 31, 2014 for patients aged 40 years and over. This database covers more than 98% of Spanish hospital admissions and includes patient’s gender and date of birth, admission and discharge dates, diagnoses at discharge (up to 14), and procedures (up to 20) performed during the hospital stay [18].

We selected patients whose medical procedures included RHA (ICD-9-MC code 81.53) and RKA (ICD-9-MC code 81.55). We stratified each cohort by diabetes status: T2DM (ICD-9-MC codes 250.x0, 250.x2) and no diabetes. Patients with type 1 diabetes were excluded (ICD-9-MC codes 250.x1, 250.x3).

We identified a total of 44,055 and 39,938 patients who underwent RHA (12.72% with T2DM) and RKA (15.01% with T2DM) over the study period. Patients who underwent RHA and RKA in the non-diabetes group were used as control groups. RHA and RKA cases were matched, one by one, with RHA controls and RKA controls, respectively, by year, age, gender and all comorbidities included in the modified Charlson comorbidity index (CCI) [19]. If more than one control was available for a case, the selection was conducted randomly. Finally, we matched 4,700 and 5,394 pairs of RHA and RKA patients, respectively, which represent 83.89% and 89.95% of the total T2DM populations.

We retrieved data about risk factors, emergency room admission, causes of revision and specific IHPC recorded during the hospitalization for RHA and RKA in any diagnosis position. The conditions studied and the codes used to identify them according to the ICD-9-MC are shown in Table 1. We considered “*any in-hospital complication*” the presence of one or more complications in any diagnosis position in the same patient.

**Table 1:** Risk factors, causes for revision and in-hospital postoperative complications with corresponding ICD-9-CM codes.

	ICD-9-CM codes
Risk factors	
Obesity	278.xx
Hypertension	401, 401.0, 401.1, 401.9
Current smoking	305.1, V15.82
Cause of revision	
Dislocation	996.42
Fracture	996.44
Periprosthetic joint infection	996.66, 996.67
Mechanical issue or loosening	996.4, 996.41, 996.43, 996.45, 996.46, 996.47, 996.49
Postoperative Complications	
Central nervous system	997.0, 997.00, 997.01, 997.02, 997.09
Cardiac	997.1
Peripheral vascular disease	997.2
Respiratory	997.3, 997.31, 997.32, 991.39, 480–488
Gastrointestinal	997.4
Genitourinary	997.5
Hematoma/seroma	998.1, 998.11, 998.12, 998.13
Wound dehiscence	998.0, 998.00, 998.01, 998.02, 998.09, 998.3, 998.31, 998.32, 998.33
Postoperative infection	998.5, 998.51, 998.59
Deep vein thrombosis	453.4, 453.40, 453.41, 453.42
Pulmonary embolism	415.1, 415.11, 415.19
Acute post hemorrhagic anemia	285.1
Septic shock	785.52, 995.91, 995.92
Renal failure	584, 584.5–584.9
Urinary tract infection	599.0

Hospital outcome variables included LOHS and IHM, defined as the proportion of patients who died during the hospital stay.

### Statistical analysis

Descriptive statistical analyses were performed. Variables are expressed as proportions, as the means with SDs or as medians with IQRs (LOHS). We generated bivariate conditional logistic regression models to compare risk factors, causes for revision, specific IHPC and in-hospital outcomes between T2DM patients and controls in the RHA cohort and in the RKA cohort.

To assess the effect of T2DM on “*any postoperative complications”* after controlling for additional potential confounders, we generated two unconditional multivariable logistic regression models that included those variables recorded and not used to match cases and controls.

Finally, we performed two unconditional logistic regression analyses to identify the variables associated with “*any postoperative complications*” as a binary outcome among all patients with T2DM who underwent RHA (n = 5,602) and RKA (n = 5,996) before matching.

The variables included in the multivariable models were those with significant results in the bivariate analysis and those considered relevant in other investigations. Estimates were Odds Ratios (OR) with their 95% confidence intervals.

Matching cases with controls and all statistical analyses were performed with Stata version 10.1 (Stata, College Station, Texas, USA). Statistical significance was set at p<0.05 (2-tailed).

### Ethical aspects

Data confidentiality was maintained at all times in accordance with Spanish legislation. Given the anonymous and mandatory nature of the dataset, it was not deemed necessary to obtain informed consent. The study protocol was approved by the ethics committee of the Universidad Rey Juan Carlos, Madrid, Spain.

## Results

Before matching was conducted, patients with T2DM who underwent RHA (n = 5,602) were found to be significantly older (73.33±8.93 years vs. 70.85±11.33 years; p = 0.001) and had a higher mean number of coexisting medical conditions according to the CCI (0.62±0.36 vs. 0.51±0.23; p<0.001) than patients without diabetes; there were also more males in the T2DM group than in the non-T2DM group (45.62% vs. 43.31%; p<0.001) (n = 38,453).

RKA patients with T2DM (n = 5,996) were significantly older (71.84±7.23 years vs. 70.38±8.08 years) and had more comorbidities according to the CCI (0.52±0.25 vs. 0.43±0.16 p<0.001) than non-diabetic patients; there were more males in the T2DM group (29.70% vs. 27.07%; p<0.001) than in the non-diabetic patient group (n = 33,942).

Table 2 shows the characteristics of hospital admission for patients who underwent RHA and RKA after matching patients with T2DM to non-T2DM controls.

**Table 2:** Characteristics and comorbidities of patients with type 2 diabetes and non diabetic matched controls who underwent revision total hip arthroplasty and revision total knee arthroplasty in Spain, 2005–2014, according to the Charlson comorbidity index (CCI).

		Revision hip arthroplasty		Revision knee arthroplasty	
		T2DM patients	Matched controls	T2DM patients	Matched controls
Year	2005/06, n (%)	762 (16.21)	762 (16.21)	695 (12.88)	695 (12.88)
	2007/08, n (%)	815 (17.34)	815 (17.34)	909 (16.85)	909 (16.85)
	2009/10, n (%)	1008 (21.45)	1008 (21.45)	1141 (21.15)	1141 (21.15)
	2011/12, n (%)	971 (20.66)	971 (20.66)	1193 (22.12)	1193 (22.12)
	2013/14, n (%)	1144 (24.34)	1144 (24.34)	1456 (26.99)	1456 (26.99)
Female gender	n (%)	2647 (56.32)	2647 (56.32)	3894 (72.19)	3894 (72.19)
Age	mean (SD)	73.32 (8.72)	73.32 (8.72)	71.77 (7.05)	71.77 (7.05)
Age groups, years	40–64, n (%)	738 (15.7)	738 (15.7)	822 (15.24)	822 (15.24)
	65–74, n (%)	1600 (34.04)	1600 (34.04)	2480 (45.98)	2480 (45.98)
	75–84, n (%)	2051 (43.64)	2051 (43.64)	1997 (37.02)	1997 (37.02)
	≥85, n (%)	311 (6.62)	311 (6.62)	95 (1.76)	95 (1.76)
Myocardial infarction	n (%)	43 (0.91)	43 (0.91)	32 (0.59)	32 (0.59)
Congestive heart failure	n (%)	66 (1.4)	66 (1.4)	38 (0.7)	38 (0.7)
Peripheral vascular disease	n (%)	13 (0.28)	13 (0.28)	11 (0.2)	11 (0.2)
Cerebrovascular disease	n (%)	21 (0.45)	21 (0.45)	11 (0.2)	11 (0.2)
Dementia	n (%)	29 (0.62)	29 (0.62)	1 (0.02)	1 (0.02)
Chronic pulmonary disease	n (%)	358 (7.62)	358 (7.62)	399 (7.4)	399 (7.4)
Rheumatoid disease	n (%)	69 (1.47)	69 (1.47)	65 (1.21)	65 (1.21)
Peptic ulcer disease	n (%)	4 (0.09)	4 (0.09)	2 (0.04)	2 (0.04)
Mild liver disease	n (%)	41 (0.87)	41 (0.87)	29 (0.54)	29 (0.54)
Hemiplegia or paraplegia	n (%)	1 (0.02)	1 (0.02)	0 (0)	0 (0)
Renal disease	n (%)	104 (2.21)	104 (2.21)	83 (1.54)	83 (1.54)
Cancer	n (%)	20 (0.43)	20 (0.43)	13 (0.24)	13 (0.24)
Moderate/severe liver disease	n (%)	0 (0)	0 (0)	2 (0.04)	2 (0.04)
Metastatic cancer	n (%)	1 (0.02)	1 (0.02)	0 (0)	0 (0)
AIDS	n (%)	0 (0)	0 (0)	0 (0)	0 (0)

The distribution according to year of admission shows that the number of hospitalizations of patients with T2DM who underwent RHA was 762 (16.21%) in 2005/06 and increased to 1,144 (24.34%) in 2013/14. In patients who underwent RKA, the number of T2DM patients increased from 695 (12.88%) in 2005/06 to 1,456 (26.99%) in 2013/14. A total of 56.32% of patients in the RHA cohort and 72.19% of patients in the RKA cohort were women. The mean age was 73.32 years and 71.77 years for the RHA and RKA procedures, respectively.

In both procedures, among patients with T2DM, the most common comorbidities according to the CCI included chronic pulmonary diseases (7.62% for RHA and 7.40% for RKA), renal disease (2.21% for RHA and 1.54% for RKA) and rheumatoid disease (1.47% for RHA and 1.21% for RKA) (Table 2).

The risk factors, emergency room admissions, causes of revision, IHPC and hospital outcomes of patients with T2DM who underwent RHA and RKA with their matched non-T2DM controls are shown in Table 3.

**Table 3:** Risk factors, emergency room admissions, cause of revision, postoperative complications and hospital outcomes in patients with type 2 diabetes who underwent total revision hip and knee arthroplasty and their matched non-type 2 diabetes controls in Spain, 2005–2014.

	REVISION HIP ARTHROPLASTY			REVISION KNEE ARTHROPLASTY		
	No diabetes	Diabetes		No diabetes	Diabetes	
	N (%)	N (%)	p-value	N (%)	N (%)	p-value
RISK FACTORS						
Obesity	195 (4.15)	437 (9.3)	<0.001	433 (8.03)	745 (13.81)	<0.001
Hypertension	1711 (36.4)	3091 (65.77)	<0.001	2311 (42.84)	3888 (72.08)	<0.001
Current smoking	157 (3.34)	227 (4.83)	<0.001	123 (2.28)	188 (3.49)	<0.001
Emergency room admission	1337 (28.45)	1601 (34.06)	<0.001	537 (9.96)	627 (11.62)	0.005
CAUSE OF REVISION						
Dislocation	657 (13.98)	723 (15.38)	0.045	185 (3.43)	178 (3.3)	0.708
Fracture	292 (6.21)	305 (6.49)	0.576	75 (1.39)	72 (1.33)	0.802
Periprosthetic joint infection	589 (12.53)	707 (15.04)	<0.001	900 (16.69)	1040 (19.28)	<0.001
Mechanical issue or loosening	2733 (58.15)	2564 (54.55)	<0.001	2863 (53.08)	2879 (53.37)	0.755
POSTOPERATIVE COMPLICATIONS						
Central nervous system	16 (0.34)	12 (0.26)	0.451	2 (0.04)	5 (0.09)	0.273
Cardiac	23 (0.49)	22 (0.47)	0.882	21 (0.39)	22 (0.41)	0.879
Peripheral vascular disease	3 (0.06)	3 (0.06)	0.999	3 (0.06)	4 (0.07)	0.706
Respiratory	22 (0.47)	32 (0.68)	0.168	14 (0.26)	13 (0.24)	0.847
Gastrointestinal	13 (0.28)	13 (0.28)	0.999	5 (0.09)	4 (0.07)	0.739
Genitourinary	27 (0.57)	26 (0.55)	0.886	10 (0.19)	19 (0.35)	0.100
Hematoma/seroma	141 (3)	137 (2.91)	0.808	118 (2.19)	116 (2.15)	0.895
Wound dehiscence	41 (0.87)	55 (1.17)	0.155	56 (1.04)	49 (0.91)	0.495
Postoperative infection	138 (2.94)	212 (4.51)	<0.001	83 (1.54)	90 (1.67)	0.586
Deep vein thrombosis	9 (0.19)	3 (0.06)	0.099	4 (0.07)	6 (0.11)	0.530
Pulmonary embolism	4 (0.09)	3 (0.06)	0.706	5 (0.09)	5 (0.09)	0.999
Acute posthemorrhagic anemia	362 (7.7)	448 (9.53)	0.002	303 (5.62)	389 (7.21)	0.001
Septic shock	17 (0.36)	18 (0.38)	0.866	12 (0.22)	8 (0.15)	0.374
Renal failure	58 (1.23)	65 (1.38)	0.518	29 (0.54)	37 (0.69)	0.311
Urinary tract infection	94 (2)	113 (2.4)	0.177	39 (0.72)	61 (1.13)	0.029
Any in-hospital complication	735 (15.64)	893 (19)	<0.001	598 (11.09)	698 (12.94)	0.003
Hospital outcomes						
Length of hospital stay (median, IQR)	11 (8–19)	12 (8–21)	<0.001	9 (6–14)	9 (7–15)	0.120
In-hospital mortality	36 (0.77)	65 (1.38)	0.004	10 (0.19)	14 (0.26)	0.416

The P value for the difference between patients with type 2 diabetes and matched controls was calculated with the bivariate conditional logistic regression model.

In the RHA cohort, patients with T2DM had a higher incidence of obesity (9.3% vs. 4.15%, p<0.001), hypertension (65.77% vs. 36.40%, p<0.001), smoking (4.83% vs. 3.34%, p<0.001), dislocation of a prosthetic joint (15.38% vs. 13.98, p = 0.045) and infection due to internal joint prosthesis (15.04% vs. 12.53%, p<0.001) than those without diabetes. However, diabetic patients had a lower incidence of mechanical/loosening complications or internal orthopedic device implants (54.55% vs. 58.15%, p<0.001) than non-T2DM controls.

In both procedures, the most common IHPC was acute post-hemorrhagic anemia.

For patients who underwent RHA, the rates of postoperative infection (4.51% vs. 2.94%, p<0.001), acute post-hemorrhagic anemia (9.53% vs. 7.70%, p<0.001) and “*any postoperative complication*” (19% vs. 15.64%) were significantly higher in diabetic patients than in those without T2DM, as can been seen in Table 3.

In the RKA cohort, the rates of obesity (13.81% vs. 8.03%, p<0.001), hypertension (72.08% vs. 42.84%, p<0.001), smoking (3.49% vs. 2.28%, p<0.001), infection due to internal joint prosthesis (19.28% vs. 16.69%, p<0.001), acute posthemorrhagic anemia (7.21% vs. 5.62%; p = 0.001) and urinary tract infection (1.13% vs. 0.72%; p = 0.029) were significantly higher in patients with diabetes than in their matched controls. Additionally, the presence of “*any postoperative complications*” was significantly higher in patients with diabetes than in those without diabetes (12.94% vs. 11.09%; p = 0.003).

In both procedure groups, the emergency room admission rate was significantly higher in diabetic patients than in those without diabetes (34.06% vs. 28.45% in RHA and 11.62% vs. 9.96% in RKA).

In the RHA cohort, the mean LOHS was slightly but significantly higher in patients with T2DM than in non-T2DM controls (12 days vs. 11 days). No differences were found for patients who underwent RKA.

For those who underwent RHA, IHM was almost twice as high in patients with T2DM (1.38% vs. 0.77%; p = 0.004) than in matched controls. No differences were found for RKA.

The results of the unconditional logistic regression models that assessed the effect of T2DM on “*any postoperative complications”* after controlling for unmatched variables found significant differences between cases and controls, as shown in Table 4.

**Table 4:** Logistic regression analysis of the factors associated with in-hospital postoperative complications in all patients after revision hip arthroplasty and revision knee arthroplasty in Spain (2005–2014).

	OR (95%CI)
	REVISION HIP ARTHROPLASTY
Diabetes	1.14 (1.03–1.29)
Obesity	1.38 (1.13–1.68)
Hypertension	1.13 (1.01–1.26)
Current smoking	1.39 (1.09–1.78)
Emergency room admission	1.54 (1.36–1.74)
Dislocation	0.93 (0.78–1.11)
Periprosthetic joint infection	1.70 (1.45–1.98)
Mechanical issue or loosening	0.98 (0.86–1.13)
	REVISION KNEE ARTHROPLASTY
Diabetes	1.15 (1.02–1.30)
Obesity	1.53 (1.29–1.81)
Hypertension	0.95 (0.84–1.08)
Current smoking	0.94 **(0.**67–1.34)
Emergency room admission	1.79 (1.50–2.14)
Periprosthetic joint infection	1.53 (1.28–1.82)
Mechanical issue or loosening	1.13 (0.98–1.30)

OR: Odds Ratio. 95%CI: 95% confidence interval. Variables included in the revision hip arthroplasty model were: Diabetes; Obesity; Hypertension; Current smoking; Emergency room admission; Dislocation; Fracture; Periprosthetic joint infection; Mechanical issue or loosening. Variables included in the knee arthroplasty model were: Diabetes; Obesity; Hypertension; Current smoking; Emergency room admission; Dislocation; Fracture; Periprosthetic joint infection; Mechanical issue or loosening.

As seen in the table, when all significant variables in Table 3 were included in the model, diabetes remained a significant risk factor for in-hospital postoperative complications for either RHA and RKA with ORs of 1.14 (95%CI 1.03–1.29) and 1.15 (1.02–1.30), respectively.

Table 5 shows the results of logistic regression analyses to assess the factors associated with “*any in-hospital postoperative complications*” in patients with T2DM during the hospital admission for RHA and RKA procedures.

**Table 5:** Logistic regression analysis of the factors associated with in-hospital postoperative complications in all patients with type 2 diabetes after revision hip arthroplasty and revision knee arthroplasty in Spain (2005–2014).

		OR (95%CI)
		REVISION HIP ARTHROPLASTY (n = 5,602)
Gender	Female	1.07 (0.93–1.23)
Age	40–64 years	1
	65–74 years	0.88 (0.71–1.09)
	75–84 years	1.25 (1.02–1.53)
	85 or over	1.60 (1.20–2.13)
Obesity	Yes	1.33 (1.08–1.66)
Periprosthetic joint infection	Yes	1.58 (1.33–1.87)
Myocardial infarction	Yes	1.72 (1.19–2.50)
Congestive heart failure	Yes	2.65 (1.99–3.53)
Cerebrovascular disease	Yes	2.21 (1.52–3.23)
Mild liver disease	Yes	1.47 (1.03–2.09)
Renal disease	Yes	1.61 (1.25–2.08)
Emergency room admission	Yes	1.44 (1.25–1.65)
Year		0.94 (0.89–0.98)
		REVISION KNEE ARTHROPLASTY (n = 5,996)
Gender	Female	1.00 (0.85–1.18)
Age	40–64 years	1
	65–74 years	1.39 (1.09–1.77)
	75–84 years	1.63 (1.27–2.09)
	85 or over	2.84 (1.81–4.48)
Obesity	Yes	1.32 (1.09–1.61)
Periprosthetic joint infection	Yes	1.48 (1.23–1.79)
Myocardial infarction	Yes	1.72 (1.07–2.77)
Congestive heart failure	Yes	2.20 (1.49–3.24)
Mild liver disease	Yes	1.77 (1.09–2.85)
Renal disease	Yes	1.56 (1.12–2.19)
Emergency room admission	Yes	1.46 (1.17–1.82)
Year		0.97 (0.92–1.08)

OR: Odds Ratio. 95%CI: 95% confidence interval. Only variables that showed a significant association are shown.

Older age, obesity, infection due to internal joint prosthesis, myocardial infarction, congestive heart failure, mild liver disease and renal disease increase the risk of in-hospital postoperative complications for T2DM patients who underwent RHA and RKA.

Among T2DM patients who underwent RHA, cerebrovascular disease was also associated with a higher risk of in-hospital postoperative complications.

Over the entire study period, a diabetic patient hospitalized for RHA through the emergency room (ER) was 1.44 (95%CI, 1.25–1.65) times more likely to have any in-hospital postoperative complications than a diabetic patient admitted electively. We also found a significant effect of ER admission among T2DM patients who underwent RKA (OR, 1.46; 95%CI, 1.17–1.82).

The risk of IHPC for diabetic patients who underwent RHA decreased significantly from 2005 to 2014 after multivariable adjustment (OR 0.94; 95%CI, 0.89–0.98). The corresponding crude prevalences of IHPC were 17.43% in 2005 and 15.29% in year 2014. For RKA, the adjusted OR was not significant (0.97; 95%CI 0.92–1.08), with values of 9.97% and 10.40% in 2005 and 2014, respectively.

## Discussion

T2DM is considered a common morbidity that correlates with poor clinical postoperative outcomes after orthopedic surgery [7, 20]. According to information obtained from the CMBD, compared with nondiabetic counterparts, patients with T2DM who underwent either revision hip or knee arthroplasty had a higher frequency of overall postoperative complications, specifically acute posthemorrhagic anemia, compared with patients without diabetes.

It has been shown that diabetes increases the risk of postoperative complications in the surgical setting [21]. We found that significantly more “*any postoperative complications*” existed after revised joint arthroplasty in patients with diabetes than in those without diabetes after controlling for possible confounders. However, limited and contradictory evidence exists regarding orthopedic surgery, with some studies showing that a history of diabetes increases postoperative complication risk [22], while other studies do not [18]. Ekinci et al. [23] concluded that patients with well-controlled diabetes (median HbA1c 6.9% [IQR 6.4–7.7]) have mitigated the risks associated with diabetes in surgical settings.

Several studies have reported that anemia is an important risk factor associated with perioperative complications and prolonged hospital stay following RHA and RKA and is also a predisposing factor for periprosthetic joint infections [5, 24]. Again, anemia is a common disease found in patients with poorly controlled diabetes and diabetic patients with nephropathy [25]. The inherent risk of anemia found in T2DM patients should be taken into account during preoperative risk management.

The presence of diabetes has been described as a risk factor for infection after total hip arthroplasty [26], in line with our results in the RHA cohort. We have found that the rate of postoperative infection is higher in diabetic patients than in non-diabetic patients. A study that used the Danish Hip Arthroplasty Registry reported that the risk of infection after total hip replacement in patients with diabetes is associated with the presence of diabetes-related complications (adjusted RR, 1.36; 95%CI, 1.12–1.66), as well as a duration of diabetes for less than five years prior to surgery (adjusted RR, 1.69; 95%CI, 1.24–2.32), which is probably the result of poorly controlled diabetes and levels of glucose [8]. Good preoperative glycemic control is associated with a decrease in infectious complications after surgery (adjusted OR, 2.13; 95%CI, 1.23–3.70) [16].

We found that infection due to internal joint prosthesis was a strong predictor of IHPC in patients with T2DM after either RHA or RKA. There is a clearly established association between T2DM, periprosthetic joint infection, and increased revision rates (RR, 1.49; 95% CI, 1.02–2.18), especially when patients have poor glycemic control [8]. A recent meta-analysis found that diabetes was associated with a 1.74 (95%CI, 1.45–2.09) increased risk of periprosthetic joint infection after total joint arthroplasty [27]. The level of glycemic control has also been reported to influence the risk of periprosthetic joint infection [28].

We also found that T2DM patients who underwent RKA were more likely to suffer from urinary tract infections than patients without diabetes. The relationship between diabetes and urinary tract infection has been reported by some authors; they found that diabetes is an independent factor of more urinary tract infection and reported an adjusted OR of 1.54 (95% CI, 1.47–1.60) for T2DM patients [29] which is associated with a greater cost for treatment and hospitalizations [30].

Obesity was associated with an increased risk of infection after total joint arthroplasty, in accord with previous studies [31,32]. In our study, obesity was a predictor of IHPC in patients with diabetes in both revision procedure groups. Being overweight or obese is associated with higher rates of re-revision, reoperation and periprosthetic joint infection after RKA [33]. Lubbeke et al. [34] reported that obesity has been associated with lower clinical outcome scores (surgical site infection, adjusted HR 4.1; 95% CI, 1.1–15.0 and for dislocation, 3.5; 95%CI, 1.3–9.3) when compared with non-obese patients after RHA. These findings are probably due to the presence of more difficult anatomic conditions.

Our study also highlights that older age and comorbidity were strong predictors of IHPC in patients with T2DM who underwent revision joint procedures. Myocardial infarction, congestive heart failure, mild liver disease and renal disease increase the risk of IHPC. These findings are consistent with those of previous studies [17, 35–37]. Koenig et al. [36] found that both age and CCI were independent predictors of major adverse events after RHA (adjusted OR, 1.04; 95%CI, 1.00–1.07; and adjusted OR, 1.22; 95%CI, 1.03–1.45, respectively). In a case-control study, Basilico et al. [37], reported that patients undergoing a revision joint procedure were 2.2 times more likely to have a cardiac complication (95% CI, 1.2–3.9), and increased age was also a risk factor (OR, 1.7; 95% CI, 0.9–3.4).

Various studies have shown that patients undergoing emergent RHA will have a more complex hospitalization and will require more assistance at discharge compared to patients who undergo elective RHA [38, 39]. Sams et al. [39] concluded that RHA performed emergently adds an additional layer of complexity to the case and may have a significant financial and clinical impact on this population and the institutions that provide care. As expected, our data highlight that emergency room admission is a predictor of IHPC in patients with T2DM after revision joint arthroplasty.

This study also uncovers a decrease in the probability of suffering from “*any postoperative complication*” from 2005 to 2014 in T2DM patients who underwent RHA. One explanation is that orthopedic surgeons may prefer to operate on patients with well-controlled diabetes and the control of diabetes is associated with less IHPC [8]. In Spain, Villanueva-Martinez et al. [17] found a significant decrease in LOHS from 20.6 days in 2001 to 19.1 days in 2008 (p<0.001) in a population based study that included 32,280 cases of RHA and suggested that the presence of more specialized departments that more efficiently rehabilitated and discharged patients and an increased rate of discharges to short and long-term care facilities may be associated with a decrease in LOS. Strict adhesion to international protocols may also help to reduce specific complications such as periprosthetic joint infections [40]. These reasons may explain our results.

The strengths of our study included its large simple size, standardized methodology, and use of year-, gender- age- and comorbidity- matched non-T2DM patients to control for the confounding effects of these variables. However, our study had several limitations. First, the database was designed for administrative rather than research purposes, and conditions such as the nature of infection, hypertension, obesity and smoking may not have been adequately recorded in the database [18].

Administrative databases can be affected by selection and information bias and confounding [41–3]. It has been found that for diseases that can be treated in general practice, cases included in the hospital discharge databases to some degree represent a selected patient group, with either high severity of the disease or severe comorbidity leading to a lower threshold for hospital admission compared with patients without comorbidity [41]. However, these bias would not affect surgical procedures like revision hip and knee arthroplasty.

Regarding information bias, it has been described that private clinics and hospitals are possible sources of underreporting [41, 43]. Differences in registration practice among hospital departments and over time are another potential limitation [41, 43]. Also the lack of information on the severity of diseases or deficiencies in the precision of ICD codes definitions (including post-operative complications) can affect the validity of the results [41–43].

Regarding confounding incomplete registration of some diagnoses and missing data on other characteristics (eg, lifestyle risk factors) may leave substantial residual and unmeasured confounding [41].

Unfortunately, Spain does not have a national arthroplasty registry. In the absence of such a registry, the administrative databases can provide alternative information sources to describe and analyze the trends and characteristics of joint arthroplasty at a national level [7, 12, 44, 45].

Second, the database that we used contained no information about the type of revision (one vs. two-stage procedure in infected cases), the surgical technique (most total hip revisions are performed without cement and most total knee revisions are performed with antibiotic loaded cement in our country, thus reducing the rate of infections) [40] and did not offer information regarding the exact reason for the revision and length of time between the primary procedure and the revision, which may have affected our outcome variables.

Third, the validity of diabetes as a discharge diagnosis in the Spanish National Hospital Discharge Database has not been evaluated. However, Khokhar et al. reviewed 18 validation studies of administrative data for the definition of diabetes and found that sensitivity ranged from 51.78% to 100%, specificity ranged from 88% to 100%, positive predictive value ranged from 21% to 99%, and negative predictive value ranged from 60.32% to 99.63% [46]. These studies were conducted in Canada/Australia which cannot be generalized to Spain. These authors concluded that overall, administrative health databases are useful for diabetes surveillance [46]. More recently, in Canada, Jiang and colleagues matched 63,483 patients between the APPROACH database and the Discharge Abstract Database in terms of diabetes and found that the validity of the diabetes discharge diagnosis mostly remained consistent over time. Between 2002 and 2013, sensitivity, specificity, positive predictive value and negative predictive value ranged from 81% to 98% for diabetes and the corresponding kappa index scores ranged from 0.80 to 0.89. No significant differences in the validity of coding were found across age, gender or hospital location subgroups [47]. The only study conducted in Spain by Ribera et al found similar figures with a sensitivity of 55% and 97% specificity [43]. The only moderate sensitivity means that an important proportion of T2DM patients don’t have this diagnosis codified in their discharge report [43]. On the other hand, the very high specificity means that most patients without T2DM diagnosis don’t really have this disease, therefore we think the effect of this misclassification on our case-control design is possibly very small.

Studies conducted in Spain and other countries have used hospital discharge data to assess the effect of diabetes on the outcomes of hip and knee arthroplasty [7, 12].

Finally, we lacked data on specific characteristic of T2DM such as glycosylated hemoglobin measurements, blood glucose levels, use of insulin or antidiabetic drugs or duration of the disease, therefore we cannot assess the effect of diabetes control on our outcome variables.

In conclusion, this study highlights that after controlling for the effects of possible confounder variables, patients with T2DM who undergo RHA and RKA have more risk factors and a higher frequency of overall IHPC, specifically acute posthemorrhagic anemia, urinary tract infection and postoperative infection, than non-T2DM patients. Among RHA patients, LOHS and IHM were higher in patients with diabetes. Older age and comorbidities such as myocardial infarction, congestive heart failure, mild liver disease and renal disease were strong predictors of IHPC after revision joint arthroplasty in patients with T2DM. For both procedures, emergency room admission was a predictor of IHPC in diabetic patients. Among diabetic patients who underwent RHA, a significant decrease in IHPC was found between 2005 and 2014.

## Abbreviations

CCI: Charlson comorbidity index
ICD-9-CM: International Classification of Diseases-Ninth Revision, Clinical Modification
IHPC: in-hospital postoperative complications
IHM: in-hospital mortality
LOHS: length of stay
RHA: revisión of total hip arthroplasty
RKA: revision of total knee arthroplasty
T2DM: type 2 diabetes
